# Nutritional and non-nutritional factors associated with low birth weight in Sawula Town, Gamo Gofa Zone, Southern Ethiopia

**DOI:** 10.1186/s13104-019-4529-0

**Published:** 2019-08-23

**Authors:** Zelalem Abera, Daba Ejara, Samson Gebremedhin

**Affiliations:** 1 JSI/Transform Primary Health Care Project, Arba Minch, Ethiopia; 2 Mada Walabu University, Shashamene campus, Shashamene, Ethiopia; 3 St Paul Hospital, Millennium Medical College, Addis Ababa, Ethiopia

**Keywords:** Low birth weight, Risk factors, Pregnant women, Sawula, Southern ethiopia

## Abstract

**Objective:**

Birth weight is a major predictor of infant growth and survival, and is dependent on maternal health and nutrition during pregnancy. This study aimed to determine the magnitude and identify nutritional and non-nutritional factors associated with LBW among newborn in Southern Ethiopia. Institutional-based cross-sectional study was used. Systematic random sampling was employed to select the study subjects. Data were entered into Epi-info Version 3.5.3 and then exported to SPSS Version 20 for analysis. Bivariable and multivariable logistic regression were used to compare birth weight across categories of independent variables. The output of the analysis were presented using adjusted odd ratio (AOR) with the corresponding 95% confidence interval (CI).

**Results:**

A total of 358 mothers participated in the study. The mean (± standard deviation) birth weight of all term infants was 3304 (± 684) gram. The prevalence of LBW was 17.3% (95% CI 13.7–21.2%). Mothers who had MUAC less than 23 cm [AOR = 6.51 (95% CI 2.85–14.91)] and with hemoglobin < 11 mg/dl [AOR = 3.42 (95% CI 1.73–6.78)] have increased odds of delivering LBW and mothers who often take dairy products [AOR = 0.36 (95% CI 0.13–0.98)] were less likely deliver LBW babies than their counterparts.

## Introduction

Birth weight is defined as the weight of the newborn, measured within the first hour of life, before significant postnatal weight loss occurs. Low birth weight (LBW) is defined by World Health Organization (WHO) as a birth weight less than 2500 g [1]. According to UNICEF and WHO reports more than 20 million infants worldwide, representing 15.5% of all births are born with LBW. The level of LBW in the developing countries (16.5%) is more than double the level in the developed regions (7%). LBW levels in sub-Saharan Africa are around 13% to 15%, with little variation across the region as a whole. These rates are higher than in most other sub regions in the world, presenting a major challenge [2].

A child’s weight at birth is the most important determinant of perinatal and infant mortality and morbidity and may have an influence on health in adult life. Based on international epidemiological observations infants weighing less than 2500 g are approximately 20 times more likely to die than heavier babies. Survived LBW babies are also more likely to suffer a high incidence of malnutrition, diarrhea, infection, neurodevelopment problems and physical defects. LBW babies are at high risk of developing chronic adult disease, such as type II diabetes, hypertension and cardiovascular disease later in their adulthood life [2–6].

Ethiopian health and demography survey (EDHS) conducted in 2011 showed that among children born in the 5 years before the survey, 11% weighed less than 2.5 kilograms [7]. Studies conducted at Gonder, Jimma, and Sidama reported that the prevalence of LBW was 11.2%, 22.5% and 16.5% respectively [8-10].

As birth weight determines the future health and health related factors of the newborn, intervening before and after pregnancy in appropriate maternal nutritional and non-nutritional factors is a window of opportunity for action.

## Main text

### Methods

#### Study setting

The study was conducted in Sawula town, Gamo Gofa zone, Southern Ethiopia. Sawula town is located 505 km away from the capital Addis Ababa and 285 km far from the regional capital Hawassa. The total population of the town is 43,639. The town has one district hospital, one comprehensive health center and four health posts. There are 5272 expected deliveries in respective health institutions which was calculated based on national conversion factor for estimated deliveries per year.

#### Study design and population

Facility-based cross-sectional study was conducted from January to May 2016 in the health facilities of Sawula town. The study population was newborns of mothers who gave birth at Sawula district hospital and Sawula health center during the study period. Pregnant mothers with singleton live birth and aged greater than 15 years were included and mothers with preterm birth were excluded from the study.

#### Sample size and sampling technique

An independent sample size was calculated for both specific objectives. Accordingly, single population proportion formula was used to calculate sample for determining magnitude of LBW. In the calculation, 95% confidence level, expected proportion of 16.5% [10], 4% margin of error and 5% compensation for possible non-response were assumed. To identify factors, sample size was calculated using Epi-info software for cross-sectional study. Then the largest sample was taken to answer both objectives. Accordingly, a sample of 380 was included in the current study. There are 2636 expected deliveries in 6 months. Proportion to size allocation was carried out to allocate a total sample size for two health institutions providing delivery service in Sawula town. Systematic random sampling method was employed to select individual at (N/n = Kth) to completed the data collection within half a year, and randomly the first birth selected from 1 to 7 individual and continued until the final sample size reached based on inclusion criteria regardless of the mode of delivery.

#### Data collection procedure

Interviewer administered pretested questionnaire was used to collect the data. The questionnaire was prepared in English, then translated into the local language Amharic, and back translated to English in order to check its consistency.

Maternal height was measured to the nearest 0.1 cm using ANC DETECTO® medical scale with sliding head metallic bar. The measurement was made without shoes, standing erect, feet together, heels, buttocks, and occiput touching the stadiometer and looking straight ahead. To proxy maternal nutritional status MUAC was used. It was measured to the nearest 0.1 cm using standard non-stretchable MUAC tape and taken on the middle left arm at relaxed position without any clothing and with optimal tape tension. Birth weight was measured using pretested and calibrated digital CROWN®baby weight scale as naked body. Birth weight was measured in the first 6 h right after birth and recorded to the nearest 100 g. All anthropometric measurements were taken in duplicates and the average of the two observations was ultimately registered.

Venous blood from antecubital vein was taken for the determination of the hemoglobin and capillary blood for HIV test. Hemoglobin was determined by Sahli's haemoglobinometer (acid haematin method), and HIV test was done during labor by using HIV (1 + 2) Antibody Colloidal Gold (KHB) as a screening test, followed by HIV 1/2STAT-PAK® if positive. Where the result of STAT-PAK® is discordant with KHB, a third test, Unigold™ HIV is used as a tiebreaker to determine the result. For mothers whose HIV status known at ANC, card review was done to record HIV status of the mother on questionnaire.

#### Statistical analysis

Data were entered into Epi-info Version 3.5.3 and then exported to SPSS Version 20 for analysis. The characteristics of the study subjects were analyzed using descriptive statistics. Frequency distribution were used to summarize qualitative variables. Numerical summary measures, mean with SD, were used to summarize quantitative variables after checking for the normality of data. The magnitude of LBW was estimated using proportion with 95% CI. Binary logistic regression was used to identify factors associated with LBW. Simple logistic regression analysis was used to identify candidate variables for multiple regression. At this, p-value < 0.25 was used as a rule of thumb for screening variables. Multiple regression was performed to determine the independent association between explanatory and outcome variable after controlling for the effect of confounding. The magnitude of association was measured using OR with 95% CI. The significance of association was declared at p-value < 0.05. Before declaring the association, the model fitness was checked using Hosmer and Lemeshow goodness-of-fit test statistic.

### Results

#### Socio-demographic characteristics

Out of the total 380 sampled mothers who gave birth at two government owned health facilities in Sawula town, 358 participants responded to the questionnaire. This makes the response rate 94.4%. The mean (± SD) age of mothers is 26.9 (± 6.2) years and the highest proportion of respondents, 36.2% were between the age group 15–24 years. Most of mothers, 97.8% were married. About 75.5% were living in the family size of below five. Regarding place of residence, 56.4% of the respondents were living in rural area. Nearly half of the respondent mothers, 51.1% were illiterate and regarding their religion, 37.4% were protestants and 36.6% were Orthodox Christian (Table 1).

**Table 1:** Socio-demographic characteristics of mothers in Sawula town, Southern Ethiopia, 2016

Variables	Frequency	Percentage
Age in years (n = 356)		
15–24	129	36.2
25–34	170	47.8
35 and above	57	16.0
Mothers education status (n = 358)		
Illiterate	183	51.1
Informal education (read or write)	72	20.1
Primary education (1–8 Grade)	17	4.7
Secondary education (9–12 Grade)	34	9.5
Tertiary education	52	14.5
Family size (n = 358)		
Below five	271	75.7
Five and above	87	24.3
Place of residence (n = 358)		
Rural	202	56.4
Urban	156	43.6
Religion (n = 358)		
Protestant	134	37.4
Orthodox	131	36.6
Muslim	68	19.0
Others	25	6.9
Occupation of the mothers (n = 357)		
House wife	198	55.3
Government or non-government organization employ	50	14.0
Petty trade	43	12.0
Farmer	26	7.3
Student	24	6.7
Others	16	4.5
Marital status (n = 358)		
Married	350	97.8
Divorced	5	1.4
Single	3	0.8
Husband education status (n = 358)		
Illiterate	178	49.6
Informal education (read and write)	84	23.5
Grade education 1–8	13	3.6
Grade education 9–12	22	6.2
Tertiary education	61	17.1

#### Reproductive, obstetric and nutrition related characteristics

Of 358 mothers, 95.6% had at least one antenatal care (ANC) visit during the index pregnancy, of which 43.5% started the follow up in the first trimester. Nearly one third of deliveries were primipara.

Among study participants, 14.0% and 2.2% had pregnancy induced hypertension (PIH), and gestational diabetes mellitus, respectively. Additionally, from 357 mothers screened for HIV status during pregnancy and labor, 1.7% were HIV positive. From 358 pregnant mothers screened for hemoglobin level at admission for labor 18.4% had Hgb level less than 11 mg/dl.

Out of 358 postnatal mothers, 85.4% of them received nutrition education from health care providers during ANC visit they had in the index pregnancy. Of which 44.5% of mothers had practiced additional meal intake in the index pregnancy. Only 27.7% of mothers took iron tablets and 20.2% of mothers had deworming history in this pregnancy. Among participants, 45.3% of mothers were thin, MUAC less than 23 cm (Table 2).

**Table 2:** Reproductive, obstetric and nutritional characteristics of pregnant women in Sawula town, Southern Ethiopia, 2016

Variables	Frequency	Percentage
Sex of newborn (n = 358)		
Male	224	62.6
Female	134	37.4
Parity (n = 358)		
Primipara	122	34.1
Multipara	236	65.9
ANC (n = 358)		
Yes	343	95.8
No	15	4.2
Frequency of ANC follow up (n = 343)		
Once	29	8.4
Two times	34	9.9
Three times	144	41.9
Four or above	136	39.6
Pregnancy induced hypertension (n = 358)		
No	350	97.8
Yes	8	2.2
Gestational diabetic mellitus (n = 358)		
No	350	97.8
Yes	8	2.2
Hyperemesis gravidarum (n = 358)		
No	282	78.8
Yes	76	21.2
Hemoglobin (n = 358)		
Normal (≥ 11 g/dl)	292	81.6
Low (< 11 mg/dl)	66	18.4
HIV test status (n = 357)		
Negative	351	98.3
Positives	6	1.7
Alcohol intake during pregnancy (n = 358)		
No	351	98.0
Yes	7	2.0
Nutritional advise received during the pregnancy (n = 358)		
Yes	299	85.4
No	51	14.6
Additional meal taken during the pregnancy (n = 358)		
Yes	160	44.7
No	198	55.3
Frequency of taking dairy products		
Often	261	72.9
Sometimes	64	17.9
Seldom	33	9.2
Number of additional meal in a day (n = 160)		
One meal	80	50.0
Two meal	73	45.6
Three and above meal	7	4.4
Deworming during the current pregnancy (n = 358)		
Yes	72	20.2
No	284	79.8
Iron tablet received (n = 357)		
Yes	99	27.7
No	258	72.3
Duration of iron tablet taken (n = 99)		
Less or equals 3 months	42	42.4
Greater than 3 months	57	57.6
History of night blindness in the current pregnancy (n = 357)		
Yes	9	2.5
No	348	97.5
Maternal MUAC		
≤ 23 cm	162	45.3
> 23 cm	196	54.7

#### Prevalence of low birth weight

The mean (± SD) birth weight of the all term infants was 3304 (± 684) g. The prevalence of LBW was 17.3% (95% CI 13.7, 21.2%).

#### Factors associated with birth weight

After bivariable, multivariable logistic regression was considered to determine independent predictors on LBW. In multivariable analysis, hemoglobin level [AOR = 3.42 (95% CI 1.73–6.78)], consumption of diary product [AOR = 0.36 (95% CI 0.13–0.98)] and MUAC [AOR = 6.51 (95% CI 2.85–14.91)] had significant association (p < 0.05) with LBW (Table 3).

**Table 3:** Factors associated with low birth weight in Sawula town, Southern Ethiopia, 2016

Variables	Birth weight		Odds ratio (95% confidence interval)	
	Low	Normal	Crude	Adjusted
Age of mothers (years)				
15–24	25	104	2.50 (0.90, 6.90)	1.94 (0.68, 5.58)
25–34	32	139	2.41 (0.89, 6.52)	1.88 (0.67, 5.21)
35 and above	5	52	1^r^	1^r^
Residence				
Rural	27	175	0.53 (0.31, 0.93)*	0.89 (0.39, 2.10)
Urban	35	121	1^r^	1^r^
Maternal education				
Illiterate	23	160	0.39 (0.18, 0.83)*	0.50 (0.15, 1.73)
Formal education	25	98	0.69 (0.33, 1.47)	0.52 (0.19, 1.37)
Tertiary education	14	38	1^r^	1^r^
Education level of husband				
Illiterate	20	157	0.48 (0.22, 1.04)	0.48 (0.22, 1.03)
Formal education	29	90	1.22 (0.58, 2.25)	1.20 (0.57, 2.53)
Tertiary education	13	49	1^r^	1^r^
Wealth index				
Poorest	12	59	0.59 (0.26, 1.32)	0.72 (0.31, 1.67)
Poorer	12	59	0.59 (0.26, 1.32)	0.62 (0.27, 1.41)
Middle	11	73	0.44 (0.19, 0.99)*	0.55 (0.23, 1.29)
Richer	7	45	0.45 (0.18, 1.16)	0.62 (0.23, 1.69)
Richest	20	58	1r	1^r^
Hemoglobin (mg/dl)				
Hg < 11 mg/dl	29	37	6.15 (3.35, 11.27)*	3.42 (1.73, 6.78)*
Hg > 11 Mg/dl	33	259	1^r^	1^r^
Height of mothers				
Less than 150 cm	33	46	6.18 (3.42, 11.15)*	1.79 (0.88, 3.60)
Greater or equals 150 cm	29	250	1^r^	1^r^
Additional meal at pregnancy				
Yes	16	143	0.37 (0.20, 0.68)*	0.78 (0.33, 1.85)
No	46	152	1^r^	1^r^
Deworming at pregnancy				
Yes	3	69	0.16 (0.05, 0.55)*	0.55 (0.14, 2.12)
No	58	226	1^r^	1^r^
Maternal MUAC				
Thin	54	108	11.75 (5.39, 25.61)*	6.51 (2.85, 14.91)*
Normal	8	188	1^r^	1^r^
Parity				
Primipara	30	92	2.07 (1.19, 3.62)*	1.87 (0.97, 3.59)
Multipara	32	204	1^r^	1^r^
Frequency of ANC follow up				
Not attended at all	7	27	2.19 (0.82, 5.83)	0.88 (0.27, 2.92)
Inadequate	39	134	2.46 (1.31, 4.61)**	0.87 (0.40, 1.93)
Adequate	16	135	1^r^	1^r^
Hyperemesis gravidurium				
Yes	19	57	1.85 (1.00, 3.41)*	1.36 (0.62, 2.99)
No	43	239	1^r^	1^r^
Frequency of taking dairy products				
Often	30	231	0.25 (0.10, 0.59)*	0.36 (0.13, 0.98)*
Sometimes	21	43	0.75 (0.35, 2.41)	0.79 (0.29, 2.09)
Seldom	13	20	1^r^	1^r^
Frequency of eating flesh foods				
Often	14	90	0.81 (0.40, 1.46)	1.27 (0.49, 3.29)
Sometimes	21	67	1.64 (0.86, 3.12)	0.75 (0.28, 2.02)
Seldom	26	136	1^r^	1^r^
Frequency of eating eggs				
Often	19	142	0.47 (0.24, 0.92)*	2.20 (0.87, 5.61)
Sometimes	21	77	0.95 (0.48, 1.88)	1.43 (0.62, 3.32)
Seldom	21	73	1^r^	1^r^

1^r^ Set as a reference category

^*^ Statistically significant association at *p*-value of 0.05

### Discussion

LBW is considered as a problem great enough to trigger public health action when its incidence exceeds 15% [11]. In this study, we found that the prevalence of LBW was 17.3%. The incidence of LBW in Gonder referral hospital, 17.1% [12] and Sidama, 16.5% [10] were consistent with this study. This finding was higher than UNICEF/WHO estimates of LBW for Ethiopia [2] and study conducted in Black Lion hospital, Ethiopia that reported prevalence of 8.4% [13]. This could be due to difference in socio-economic status and this study was conducted in relatively less urbanized population where high proportion of mothers didn’t receive iron tablets, deworming and has acute malnutrition during index pregnancy.

We found that low hemoglobin level of mother during pregnancy was one of the predictor for LBW. Findings in Addis Ababa [14] and Debrebrehan [15] Ethiopia were consistent with this result. Studies in Ghana [16] and India [17] were also in agreement with this result. Furthermore, study in Pakistan also indicated that LBW was significantly associated with low maternal hemoglobin [18]. Additionally, systematic review and meta-analysis showed that anemia was found to be significantly associated with doubling the risk of LBW [19]. Studies have found that the biological plausibility of the association between maternal anemia and LBW is not fully understood [20, 21]. However, as a result of hemodilution during pregnancy, maternal anemia may develop and then leads to fetal hypoxia. This in turn may result in reduction of oxygen and nutrients to the fetus which predispose to intrauterine growth restriction, and consequently LBW [22, 23]

One of the risk factors for LBW in this study was low maternal MUAC. This finding was in consistent with a study in Sidama, Southern Ethiopia [10] and in Kersa, Oromia, Ethiopia [24]. Systematic review of twelve longitudinal studies suggested that half of the studies looked at the association between low maternal MUAC and LBW babies, all of which were found significantly increased risk among mothers with low MUAC during pregnancy [25]. As MUAC is a proxy indicator of acute maternal nutrition during pregnancy, it has a pronounced effect on birth weight of the babies.

Maternal under nutrition due to an insufficient food supply has great effect on the growth and development of fetus. The finding in this study indicated that daily intake of dairy products at third trimester has positive influence on the birth weight of the newborn. Study in Sweden supported that low milk intake in the pregnant mother was associated with IUGR of the newborn [26]. Another study also showed that maternal milk consumption in daily base was associated with greater fetal weight gain in the third trimester of pregnancy [27]. This might be due to the fact that protein in milk play a role in fetal lean body mass and reduced risk of low birth weight. In the contrary, study in the Malaysia did not suggest such the association [28].

### Conclusion

The magnitude of LBW in this study is 17.3%. The significant predictors of LBW were maternal MUAC less than 23 cm, low hemoglobin level and frequency of daily intake of dairy products at third trimester. Strengthening quality nutrition education during ANC and improving the nutritional status of women particularly during pregnancy would reduce the occurrence of LBW. Therefore, health workers should provide routine antenatal iron tablet supplementation for as per the guideline, closely monitor maternal MUAC at antenatal visit and link cases for SFP services.

## Limitation

The cross sectional nature of the data, which makes it impossible to draw inferences about the direction of relations among study variables. As the study was facility based; it is not possible to generalize the results to a particular population as compared to population-based studies. Recall errors and social desirability bias might have also occurred in the study.

## Data Availability

The dataset analyzed during the current study is available from the corresponding author on reasonable request.

## Abbreviations

ACC/SCN: Administrative Committee on Coordination Sub-Committee on Nutrition
ANC: antenatal care
AOR: adjusted odds ratio
CI: confidence interval
COR: crud odds ratio
CSA: Central Statistic Authority
EDHS: Ethiopian Demography and Health Survey
ENGINE: Empowering New Generations to Improve Nutrition and Economic opportunities
Hgb: hemoglobin
HIV: human immunodeficiency virus
IUGR: intra uterine growth retardation
LBW: low birth weight
LMIC: low and middle income countries
MLBW: moderately low birth weight
MUAC: Mid Upper Arm Circumference
OR: odds ratio
PIH: pregnancy induced hypertension
SD: standard deviation
SFP: Supplementary Feeding Program
SPSS: Statistical Program for Social Science
UNICEF: United Nations Children's Fund
UNSCN: United Nations System Standing Committee on Nutrition
VLBW: very low birth weight
WHO: World Health Organization
